# Preparation of phosphorus-doped porous carbon for high performance supercapacitors by one-step carbonization†

**DOI:** 10.1039/d0ra02398a

**Published:** 2020-05-06

**Authors:** Guanfeng Lin, Qiong Wang, Xuan Yang, Zhenghan Cai, Yongzhi Xiong, Biao Huang

**Affiliations:** Jinshan College, Fujian Agriculture and Forestry University Fuzhou 350002 China feton.lin@hotmail.com; Key Laboratory of Bio-based Material Science & Technology (Northeast Forestry University) Haerbin 150040 China; Materials Engineering College, Fujian Agriculture and Forestry University Fuzhou 350002 China

## Abstract

Biomass-derived porous carbon has received increasing attention as an energy storage device due to its cost-effectiveness, ease of manufacture, environmental friendliness, and sustainability. In this work, phosphorus-doped porous carbon was prepared from biomass sawdust (carbon source) and a small amount of phosphoric acid (P-doping source and gas expanding agent) by one-step carbonization. For comparison, parallel studies without phosphate treatment were performed under the same conditions. Benefiting from the addition of phosphoric acid, the prepared carbon material has higher carbon yield, higher specific area and micropore volume. Due to the heteroatom doping of P in the carbon material, the optimized PC-900 sample not only exhibits high specific capacitances of 292 F g^−1^ and 169.4 F g^−1^ at current densities of 0.1 A g^−1^ and 0.5 A g^−1^, respectively, but also excellent cycle longevity (98.3% capacitance retention after 5000 cycles) in 1 M H_2_SO_4_. In addition, the supercapacitor exhibits a high energy density of 10.6 W h kg^−1^ when the power density is 224.8 W kg^−1^ at a discharge current density of 0.5 A g^−1^. This work proposes a sustainable strategy to reuse waste biomass in high-performance and green supercapacitors for advanced energy storage equipment.

## Abstract 2

P-doped porous carbon can be prepared by one-step carbonization using biomass sawdust impregnated with a small amount of phosphoric acid.

## Graphical abstract

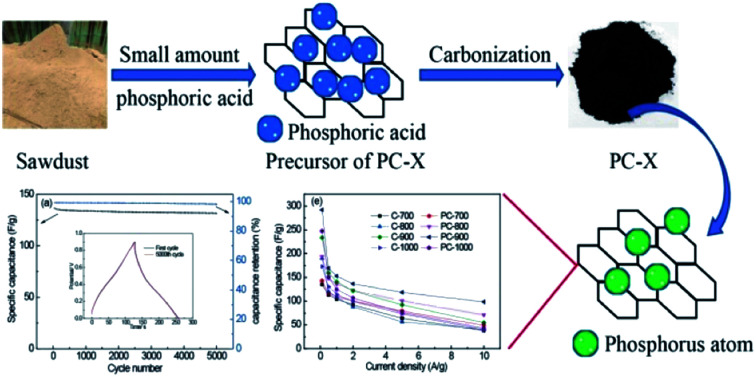

## Introduction

In recent years, due to global warming and the depletion of fossil fuels, the production of low-cost, renewable and environmentally friendly energy conversion and storage systems has become very significant.^1,2^ With the characteristics of high power density, high charge and discharge efficiency, and long cycle life, electrochemical capacitors (EC) are considered as one of the advanced energy storage systems, playing a crucial role in bridging the gap between dielectric capacitors and traditional batteries.^3–5^ Due to its high specific surface area, pore size, high physicochemical stability, electrochemical performance, and wettability, carbon-based porous materials, including carbon nanotubes,^6^ graphene,^7,8^ activated carbon,^9^ and carbide-derived carbon^10^ have been used extensively for electrode materials. There are many sources of materials for making carbon materials, such as fossil materials, polymers, and biomass.^11^ Among them, porous carbon derived from renewable biomass is widely used due to its relatively low cost, environmentally friendly characteristics, and simple synthetic process. Many studies have produced porous carbon from biomass, such as wood,^12^ bamboo,^13^ nutshells,^14^ seeds,^15^ peels,^16^ leaves,^17^ and seaweed,^18^ which have excellent electrochemical properties and can be used as electrode materials. In this case, we use fir sawdust, a low-cost and renewable forestry residue, to produce porous carbon.

As reported in the literature, pure carbon materials can provide only limited catalytically active sites, and heteroatom doping is thought to cause redistribution of charges between carbon atoms, thereby activating small molecules.^19^ Therefore, heteroatom doping (*e.g.*, O, N, P, S, B) is elected as a useful method to enhance the capacitive performance of carbon materials.^9,20–24^ Among them, the main reasons for choosing P are lower electronegativity and larger covalent radius. The lower electronegativity (2.19) of P atom compared to that of C atom (2.55) and the high electron donating property of the P atom makes the P dopant positively charged, which is advantageous for charge transfer.^20^ In addition, a much larger covalent radius of P (107 ± 3 pm) than C (73 ± 1 pm) causes many defects in the carbon material skeleton.^19^ These defects concentrate a large amount of charge, which may be the main active site. Therefore, with these unique characteristics of the P atom, P could be an ideal dopant for carbon.

At present, there have been reports of phosphorus-doped carbon as electrodes.^25–27^ However, there are only a few reports on the preparation of phosphorus-doped porous carbon electrodes using biomass as a raw material. A phosphorus-doped porous carbon, prepared using leaves as raw materials and phosphoric acid as a phosphorus dopant and its application in sodium ion and lithium ion batteries was reported by Zhu *et al.*^28^ Jiang *et al.* used NaH_2_PO_4_ as a P-doping source to prepare P-doped carbon derived from pinecone as an efficient catalyst for a Li–O_2_ battery.^19^ Nirosha *et al.* reported on preparation of phosphorus-doped carbon for asymmetric supercapacitors using *Elaeocarpus tectorius* as a carbon source and phosphoric acid as phosphorus.^29^ It is worth noting that the weight ratio of the raw material to the phosphorus dopant in the above work is relatively high, ranging from 1 : 2 to 1 : 10. In this work, in order to reduce the amount of phosphorus dopant and then reduce production cost, a small amount of phosphoric acid (the mass ratio of sawdust to phosphoric acid is 1 : 0.03) was used as a phosphorus dopant to prepare a phosphorus-doped porous carbon by one-step carbonization.

Based on these ideas, we developed a simple method for preparing P-doped porous carbon for electrode materials of supercapacitors, using sawdust and a small amount of phosphoric acid as raw materials. Sawdust is the main source of carbon. Phosphoric acid acts as a P-doping source and a gas expanding agent. To achieve high capacitance by doping phosphorus atoms into a carbon matrix, sawdust was carbonized with and without phosphoric acid at different temperatures of 700, 800, 900 and 1000 °C. A series of characterization methods were used to evaluate P-doped AC to clarify the role of phosphoric acid at different temperatures. In conclusion, this work is expected to provide new insights for the use of extremely simple methods to make heteroatom-doped materials as electrode materials for supercapacitors.

## Experimental

### Materials

Fir sawdust was crushed and sieved to obtain a particle size of 0.2 to 1 mm and dried in an oven at 110 °C for 24 h. Phosphoric acid (H_3_PO_4_, 85%) was purchased from Aladdin Industrial Co.

### Preparation of phosphorus-doped porous carbon

**Fig. 1:**
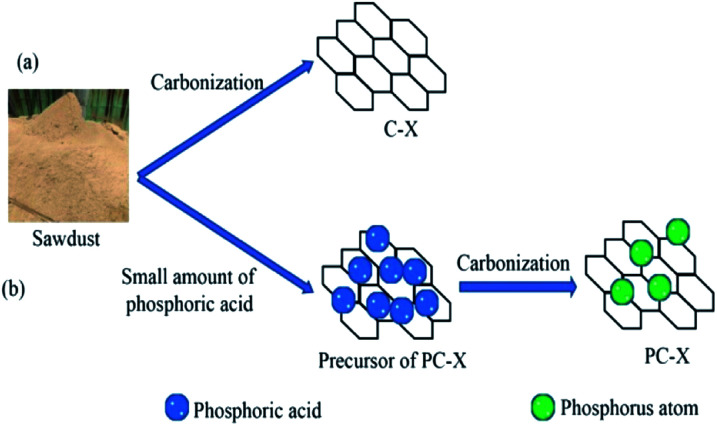
Preparation process of phosphorus-doped porous carbon.

To synthesize phosphorus-doped porous carbon, 10 g of sawdust and 30 ml of 1.0 wt% H_3_PO_4_ was mixed, and the mass ratio of sawdust to phosphoric acid was 1 : 0.03. The mixture was dried in an oven at 120 °C for 6 h. After that, the mixture was pyrolyzed in a furnace at 700, 800, 900 and 1000 °C for 4 h. For comparison, samples without H_3_PO_4_ were also prepared under the same conditions. The as-prepared sample was washed with 0.1 M hydrochloric acid and warm distilled water to neutrality, and then dried in an oven at 110 °C. Porous carbon was produced and weighted to determine its yield. Phosphorus-doped porous carbon was designated as PC-*X*, and the porous carbon prepared without H_3_PO_4_ was specified as C-*X*, where *X* represents the carbonization temperature (700, 800, 900 and 1000 °C). The strategy for preparing biomass-derived porous carbon is shown in Fig. 1.

### Characterization

N_2_ adsorption/desorption was collected by a Micromeritics (ASAP-2020) at 77 K. The specific surface area was measured by the Brunauer–Emmett–Teller (BET) method. The pore size distribution was analyzed by the density functional theory (DFT) model. The crystallinity and phase structure of the samples were evaluated by X-ray diffractometer (XRD, Philips-FEI, Netherlands) with Cu Kα radiation (*λ* = 0.1544 nm). Raman spectra were obtained on a JYHR800 Raman spectrometer, using a 514 nm laser source. Chemical composition was analyzed by X-ray photoelectron spectroscopy (XPS, Thermo Scientific ESCALAB 250Xi). The surface morphology of porous carbons was observed by a field emission scanning electron microscope (FSEM, Nova Nano SEM 230).

### Electrochemical performance

A uniform black paste was made by mixing 80 wt% porous carbon, 10 wt% acetylene black, and 10 wt% polytetrafluoroethylene (PTFE), and then coating it on a nickel foam to prepare a working electrode. The electrode was dried at 60 °C for 24 h, and compressed at 10 MPa for 1 min.

The electrochemical properties of the electrodes were evaluated in a three-electrode system, *via* VMP3 instrument (Bio-Logic, France) at room temperature. A 1 M H_2_SO_4_ aqueous solution was used as the electrolyte. In the three-electrode system, porous carbon electrode, Pt foil and Hg/HgO were used as working, counter, and reference electrodes, respectively. In the two-electrode system, both the positive and negative were the above-mentioned working electrode. Cyclic voltammetry (CV) experiments were performed at a potential range of 0–0.8 V, with a scan rate of 5 to 100 mV s^−1^. For galvanostatic charge/discharge (GCD) measurement, the current density varied within the range of 0.1–10 A g^−1^. The frequency of electrochemical impedance spectroscopy (EIS) analysis was 0.1 Hz to 10^6^ Hz.

The specific capacitance (*C*, F g^−1^) of the electrode was calculated from the GCD curve, according to the following equation:^11^

1 *C* = *I*Δ*t*/*m*Δ*V*

where *I* (A) is the discharge current, Δ*t* (s) is the discharge time, Δ*V* (V) is the potential window, and *m* (g) is the mass of the active material. The energy density and power density were calculated from the GCD curves of two-electrode system according to the following equation:^11^

2 *E* = *C* × Δ*V*^2^/(2 × 3.6)

3 *P* = *E* × 3600/Δ*t*

where *E* (W h kg^−1^) is the energy density, *P* (W kg^−1^) is the power density, C (F g^−1^) is the specific capacitance, Δ*V* (V) is the voltage change during the discharging process, and Δ*t* (s) is the discharging time.

## Results and discussion

### Preparation and characterization of phosphorus-doped porous carbon

**Fig. 2:**
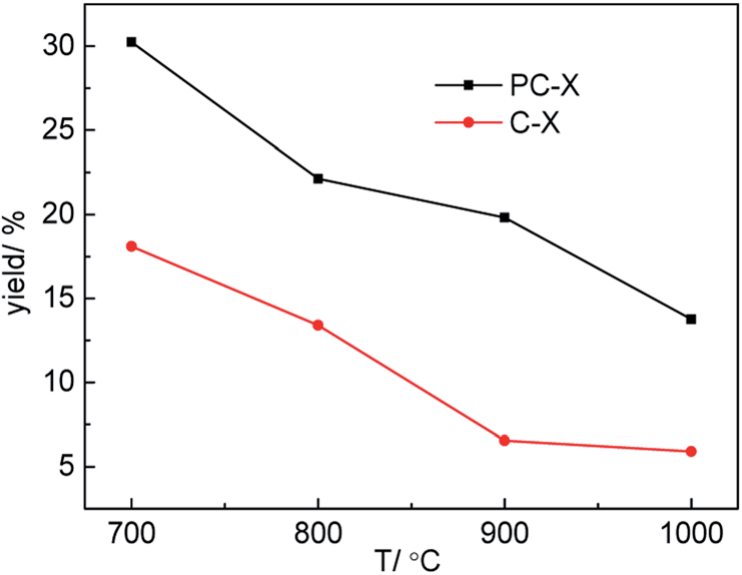
The yield of C-*X* and PC-*X*.

Carbon yield is a key factor in the preparation of porous carbon materials using various biomass derivatives. Therefore, the effect of temperature on the yield of porous carbon was discussed, and the results are shown in Fig. 2. As shown in Fig. 2, with the increase in carbonization temperature, the yields of porous carbon prepared with H_3_PO_4_ (PC-*X*) and without H_3_PO_4_ (C-*X*) decreased from 30.24% and 18.10% at 700 °C to 13.76% and 5.90% at 1000 °C, respectively. This is due to the large amount of volatile gases produced by the thermal decomposition of sawdust, especially at higher carbonization temperatures. Compared with the preparation of C-*X*, H_3_PO_4_ was incorporated into sawdust to form a phosphorous precursor of PC-*X*, and the carbon yield of the H_3_PO_4_-incorporated sample (PC-*X*) was higher than that of C-*X* due to the H_3_PO_4_ induced phosphorylation reaction in the pyrolysis process, which inhibited tar formation and protected the carbon skeleton.^30^ It was pointed out that the formation of phosphoric acid would reduce the formation of levoglucose and then enhance the yield of porous carbon.^31^ Suárez-García *et al.* also pointed out that the addition of phosphoric acid was beneficial to increase carbon yield.^32^

**Fig. 3:**
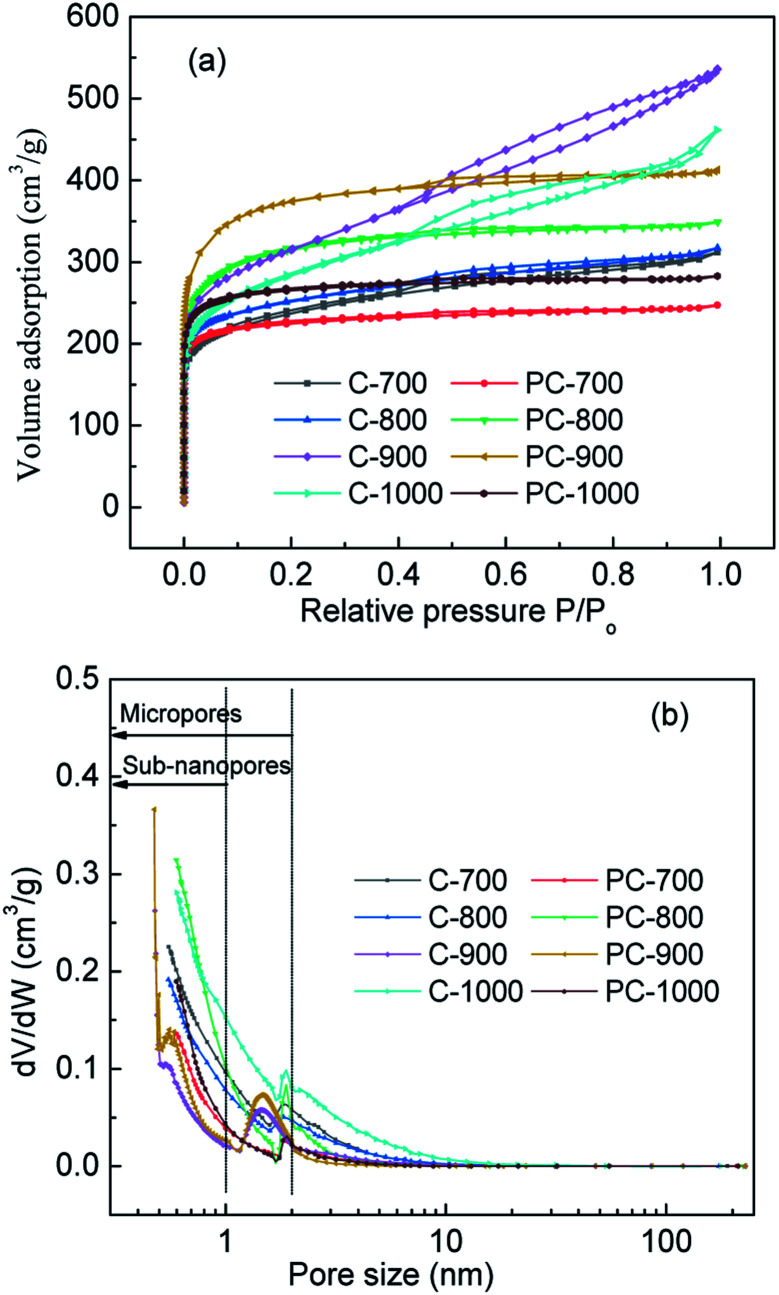
Nitrogen adsorption–desorption isotherms and pore size distribution of C-*X* and PC-*X*.

The N_2_ adsorption–desorption isotherms and pore size distribution (PSD) of samples with and without H_3_PO_4_ treatment at different carbonization temperature are presented in Fig. 3. And the pore structure parameters of the samples are presented in Table 1. For PC-*X*, all samples showed a typical type I, and the N_2_ adsorption–desorption isotherms showed a sharp increase in adsorption at *P*/*P*_o_ < 0.05, which reflects the advantages of micropores. For C-*X*, the shape of the isotherm was an I/IV type curve, and it had an obvious hysteresis loop in the high-pressure range, which could be attributed to the micropore and mesopore characteristics. At high temperatures, H_3_PO_4_ can act as a catalyst to promote bond cleavage reactions and the formation of crosslinks. Therefore, it might develop the micropores during pyrolysis. As shown in Table 1, as the carbonization temperature increases, the specific surface area (*S*_BET_) and total pore volume (*V*_tot_) of C-*X* and PC-*X* first increased and then decreased, and the *S*_BET_ and *V*_tot_ of C-900 and PC-900 reached as high as 1094.0 m^2^ g^−1^, 0.829 cm^3^ g^−1^ and 1281.6 m^2^ g^−1^, 0.638 cm^3^ g^−1^, respectively. PC-*X* samples with H_3_PO_4_ exhibited a greater *S*_BET_ and *V*_tot_ than that of C-*X* samples without H_3_PO_4_ at temperatures of 800 °C and 900 °C, indicating that a small amount of H_3_PO_4_ is an effective activated agent (gas expanding agent) at this temperature. However, when the temperature was 700 °C and 1000 °C, the *S*_BET_ and *V*_tot_ of samples with H_3_PO_4_ were smaller than C-*X* samples without H_3_PO_4_. This is due to the fact that at temperatures above 750 °C, the gas produced by partial decomposition of H_3_PO_4_, (4H_3_PO_4_ + 10C → P_4_ + 10CO + 6H_2_O) will lead to the development of pore structure.^29^ Therefore, when the temperature is lower than 750 °C, H_3_PO_4_ will not decompose, so it cannot promote the enhancement of pore structures. When the temperature is too high (1000 °C), the decomposed gas quickly escapes, destroying the preliminary structure of the activated carbon. Suárez-García *et al.* reported that the volatilization of P-compounds promoted a new increase in wider micropores and narrow micropores.^33^ It was observed that the micropore surface area (*S*_mic_) and micropore volume (*V*_mic_) of PC-*X* at the same temperature was higher than that of C-*X*, and the average pore size (*d*) showed the opposite trend, which further demonstrates that H_3_PO_4_ can develop micropores during pyrolysis. The pore size distributions of C-*X* and PC-*X* are shown in Fig. 3. The pores for C-*X* and PC-*X* were composed mainly of micropores (<2 nm), especially subnanopores (<1 nm). The main subnanopores of 0.5 to 1 nm provide accessible locations for penetration of aqueous electrolyte ions, thereby enhancing capacitance. C-*X* and PC-*X* also contained a small part of mesopores, which could be used as ion transport pathways. In addition, the mesopores of C-*X* were higher than that of PC-*X*. These results indicate that the presence of a small amount of H_3_PO_4_ is beneficial to the preparation of activated carbon with a microporous concentration.

**Table 1:** Pore parameters of C-*X* and PC-*X*

Samples	*S*_BET_ (m^2^ g^−1^)	*S*_mic_ (m^2^ g^−1^)	*V*_tot_ (cm^3^ g^−1^)	*V*_mic_ (cm^3^ g^−1^)	*D* (nm)
C-700	804.2	442.2	0.483	0.207	2.40
PC-700	743.0	603.6	0.382	0.286	2.06
C-800	844.7	548.4	0.490	0.259	2.32
PC-800	1059.2	706.1	0.540	0.330	2.04
C-900	1094.0	468.5	0.829	0.453	3.03
PC-900	1281.6	835.6	0.638	0.535	1.99
C-1000	976.6	412.6	0.713	0.191	2.92
PC-1000	875.9	725.0	0.438	0.343	2.00

**Fig. 4:**
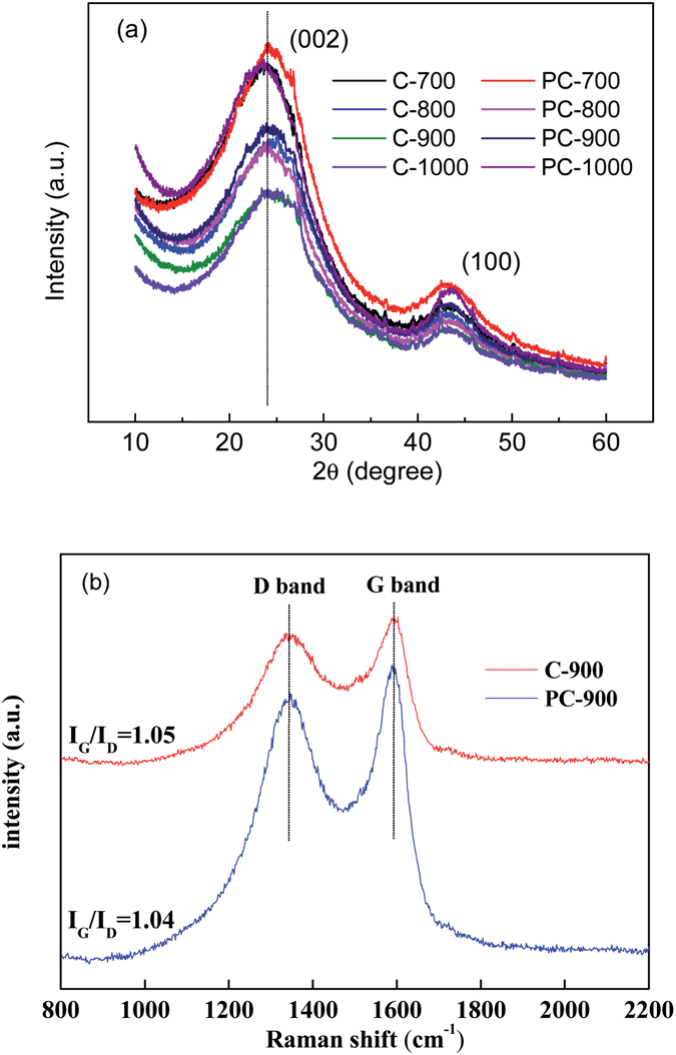
(a) XRD patterns of C-*X* and PC-*X*; (b) Raman spectra of C-900 and PC-900.

XRD patterns of C-*X* and PC-*X* are shown in Fig. 4. Two broad peaks centered at 24° and 43° can correspond to the (002) and (100) crystal planes, indicating the amorphous graphitic structure of the sample.^34^ The (002) plane of C-*X* and PC-*X* showed a shift from 26.4° of graphite to a lower value of 23.4–24.7°, which indicates an expansion in interlayer spacing. For C-*X* samples without H_3_PO_4_, as the carbonization temperature increased, its peak values (002) gradually approached 26.4°, which indicates that the increase in temperature promoted the generation of porous carbon graphitized crystallites. For PC-*X* samples with H_3_PO_4_, the peak values (002) of PC-700, PC-800, and PC-900 were almost the same, while the peak value of PC-1000 was far from 26.4°. This could be due to the flat deformation of graphite caused by the addition of a small amount of H_3_PO_4_. Interestingly, Compared to C-*X*, PC-*X* had a reduction in peak values (002) at 800, 900 and 1000 °C, especially at 1000 °C. This is because during the high-temperature heat treatment, the gas generated by the decomposition of phosphoric acid increases the interlayer spacing (consistent with the results of SEM analysis) and reduces the degree of graphitization of porous carbon.

Moreover, the higher the temperature, the faster the gas volatilizes, and the greater the interval between the carbon matrix layers. Panja *et al.* reported that P is likely to be incorporated into the edges of the graphene, which might widen the interlayer spacing.^35^ To further verify the graphitization of porous carbon with and without phosphoric acid, C-900 and PC-900 were selected for Raman spectra analysis. Fig. 4b shows the Raman spectra of C-900 and PC-900. The two discrete broad peaks around 1320 and 1580 cm^−1^ are designated as D-band and G-band, respectively, and are related to disordered carbon and sp^2^-bonded carbons.^36^ As shown in Fig. 4b, *I*_G_/*I*_D_ of C-900 was higher than that of PC-900, which implies that the degree of graphitization of P-900 is higher (consistent with the results of XRD analysis), and the addition of a small amount of H_3_PO_4_ could not only open closed pores, but also etch graphitized carbon to create a more disordered structure. In addition, the incorporation of heteroatoms in carbon atoms can cause edge defects on the carbon surface, leading to increased D-band intensity.^29^ Therefore, the PC-900 sample showed higher D-band intensity, indicating that the P-doping produced disorder.

**Fig. 5:**
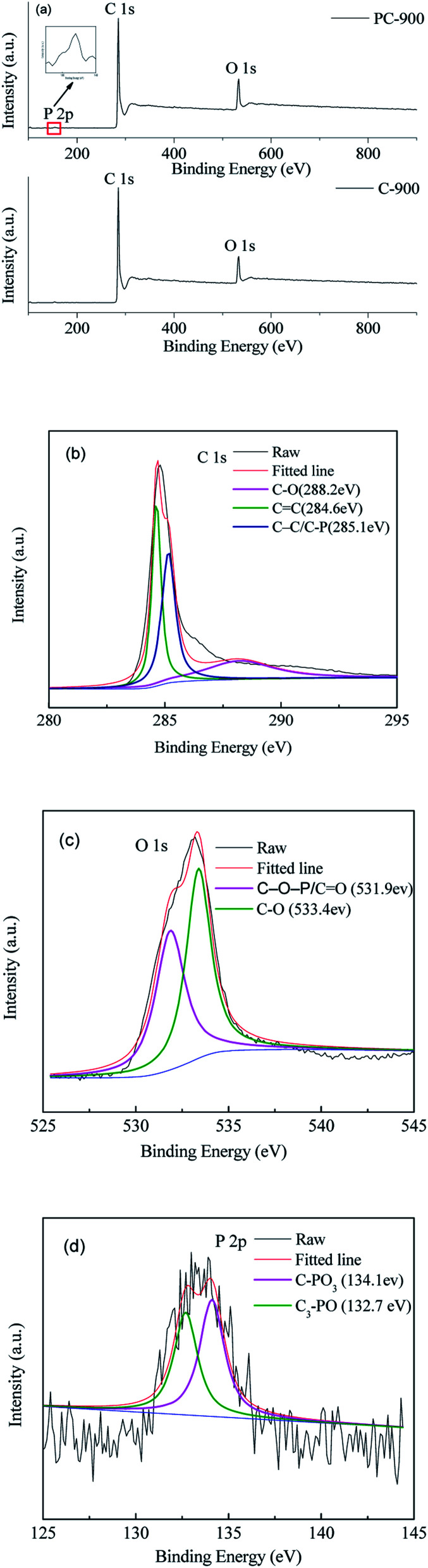
XPS spectra of C-900 and PC-900: (a) full spectrum, (b–d) deconvolution spectrum of C 1s, O 1s, and P 2p of PC-900.

The surface chemical composition of C-*X* and PC-*X* was further analyzed by XPS (Fig. 5 and S1†). As shown in Fig. 5 and S1,† the survey spectrum of C-*X* was composed mainly of C and O elements. The peak with binding energy of 284.7 and 533.3 eV represented C1s and O1s. Compared with C-*X*, PC-*X* had a tiny peak near 133.4 eV, which corresponds to P2p, indicating that adding a small amount of phosphoric acid can achieve the phosphorus doping of porous carbon. C-900 and PC-900 were performed by deconvolution analysis, and the deconvolution spectrum of C1s was divided into three main peaks, including 284.6 eV (C=C), and 285.1 eV (C–C/C–P), and at 288.2 eV (C=O).^7,8^ There were two peaks in the O1s spectrum (Fig. 5c), and the sample surface included 531.9 eV (C=O/C–O–P) and 533.4 eV (C–O).^11^ P 2p can be divided into the tetrahedral C–PO_3_ (134.1 eV) and C_3_–PO (132.7 eV).^3^ It has been reported that the incorporation of heteroatoms such as N and P will provide electrochemically active sites, enhance the wettability of the electrolyte on the electrode surface, and thus improve electrochemical performance.^29^

**Fig. 6:**
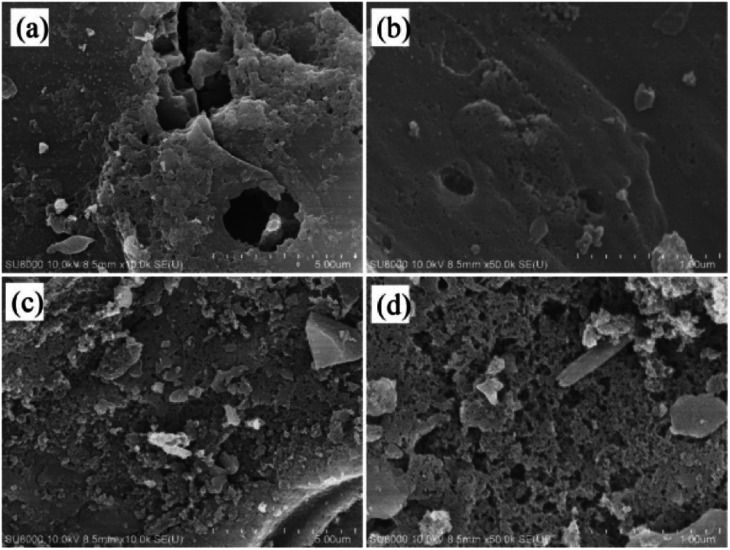
SEM images of C-900 (a and b) and PC-900 (c and d).

FE-SEM images of C-900 and PC-900 are shown in Fig. 6. The images showed the ordered porous network structure for C-900 and PC-900. Both samples had uniform pores that help promote ion migration and provide more accessible active sites in the carbon matrix. As shown in Fig. 6, PC-900 was relatively loosely arranged with many micropores in the carbon matrix. This is due to the fact that doping of phosphorus has a great influence on the morphology of the carbon matrix, which leads to the expansion of interlayer space and induces the development of pore structures. Therefore, the carbon surface provides an ionic buffer to enrich the electrolyte transport during the electrochemical process.

### Electrochemical performances of phosphorus-doped porous carbon

**Fig. 7:**
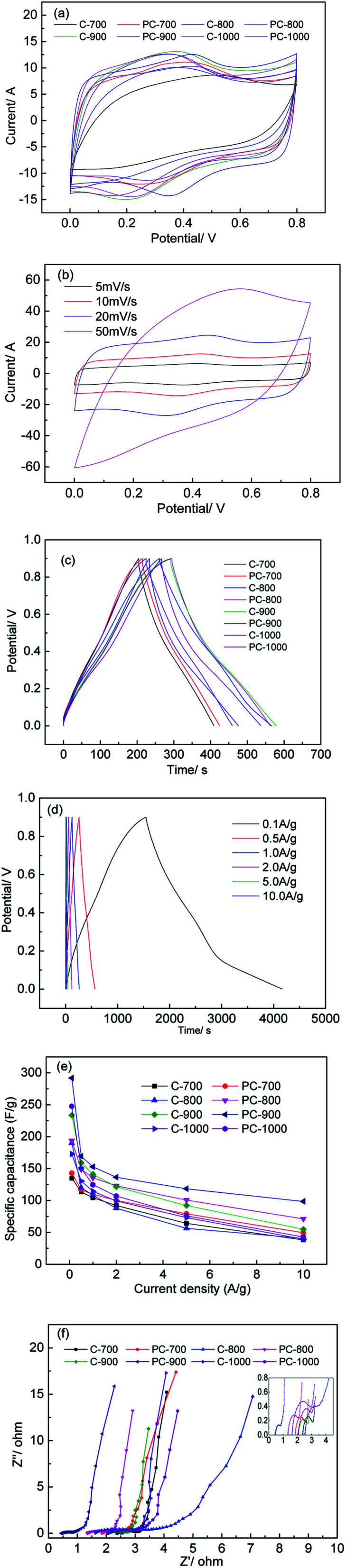
Electrochemical performance tested by a three-electrode system: (a) CV curves of C-*X* and PC-*X* at 10mV s^−1^, (b) CV curves of PC-900 at different scan rates, (c) GCD curves of C-*X* and PC-*X* at 0.5 A g^−1^, (d) GCD curves of PC-900 at different current densities, (e) the specific capacitances of C-*X* and PC-*X* at different current density, and (f) Nyquist plots of C-*X* and PC-*X*.

Fig. 7 shows the electrochemical performance of electrodes prepared with C-*X* and PC-*X* in a three-electrode system, in which a 1 M H_2_SO_4_ aqueous solution was used as the electrolyte. It was observed that all CV curves were quasirectangular and had a pair of small humps (Fig. 7a), indicating that pseudocapacitors and electric double-layer capacitors (EDLC) coexist. The samples (PC-*X*) with a small amount of H_3_PO_4_ appeared to have a much larger area at the corresponding temperature, which indicates that the capacitance is greatly enhanced. The enhancing of EDLC can be attributed to the increase in *S*_BET_ (800 and 900 °C) and the doping of phosphorus after the addition of a small amount of H_3_PO_4_. Interestingly, the *S*_BET_ of PC-700 and PC-1000 was lower than that of C-700 and C-1000, but the surrounding area in the CV curve was larger, which illustrates that adding a small amount of phosphoric acid can achieve phosphorus doping in porous carbon materials, and phosphorus-doped porous carbon can accept protons to prevent water decomposition to a certain extent, which is beneficial to improve its EDLC. It is worth noting that although *S*_BET_ is an important parameter for capacitors, not all pore structures are suitable for entering electrolyte ions. Therefore, high *S*_BET_ may increase the risk of electrolyte decomposition during charging and discharging.^18^ In addition, Guo *et al.* reported that the increased phosphorus content acts as an electron donor for carbon, causing the conduction band to shift from the Fermi level, thereby allowing electrolyte ions to be adsorbed.^37^ The electrochemical properties of C-*X* and PC-*X* are shown in Fig. 7b and S2.† As shown in Fig. 7b, even at scan rates up to 50 mV s^−1^, the CV curves of the PC-900 at different scan rates showed no obvious deformation, indicating the ideal capacitive behaviour of the electrode material. In addition, PC-*X* maintained the shape of the curve better than C-*X* at the corresponding temperature, which indicates that doping phosphorus on the surface of porous carbon will improve its EDLC. It has been reported that the introduction of P heteroatom reduces the total resistance, which helps to improve electrochemical performance.^38,39^

As shown in Fig. 7c, all GCD curves at 0.5 A g^−1^ were closed linear and presented an isosceles triangular shape, which is a typical indicator of capacitive behaviour. The discharge curve of PC-900 does not have a noticeable voltage drop, showing a small internal resistance and excellent conductivity. According to the GCD curve calculation, PC-900 showed the best performances, and the specific capacitance of the electrode material calculated at a current density of 0.5 A g^−1^ was 169 F g^−1^. Fig. 7d and S3† show the GCD curves of C-*X* and PC-*X* at different current densities. All GCD curves of C-*X* and PC-*X* electrodes were shown as linear and isosceles triangular shapes,^40^ indicating good electrochemical reversibility during charging/discharging storage. The calculated gravimetric (*C*_g_) capacitances based on the GCD results are presented in Fig. 7e. As shown in Fig. 7e, the *C*_g_ capacitance of all samples decreased with increasing current density. This might be because ions cannot diffuse into all micropores to form an electric double layer, and some faradaic reactions caused by heteroatom functional groups might not occur at high current density. At the same time, at low current density, such as PC-900, the *C*_g_ capacitance was greatly reduced.

When the current density was increased from 0.1 A g^−1^ to 0.5 A g^−1^, the *C*_g_ capacitance was reduced by 41.99%. This is due to the side reactions and irreversible redox reactions that occur at low current densities, which reduce the *C*_g_ capacitance. PC-900 provided the highest *C*_g_ capacitance of 292, 169.4, 152.8, 136.5, 118.5 and 98.5 F g^−1^ at current densities of 0.1, 0.5, 1.0, 2.0 5.0 and 10.0 A g^−1^, respectively. Table 2 presents the electrochemical performance of various biomass-derived carbons prepared by different methods. Compared to other biomass-derived carbons, we can use a small number of chemical reagents to prepare porous activated carbon with good performance, and the method is low-cost and effective.

**Table 2:** Comparison of the properties of various biomass-derived carbons

Biomass precursor	Preparation method	Mass ratio (biomass : reagent)	*S*_BET_	*C*_g_ capacitance	Measurement condition	Ref.
Eucommia ulmoides wood	Hydrothermal + H_3_PO_4_	1 : 3.3	1456	185 F g^−1^	1M H_2_SO_4_, 5mV s^−1^	41
Rubberwood	KOH	1 : 2	1932	100	1.0 m Li-TFSI, 0.1 A g^−1^	42
Rubberwood	H_3_PO_4_	1 : 1	693	129	1 M H_2_SO_4_, 1mV s^−1^	43
Firewood	KOH	1 : 1	1064	180	0.5 M H_2_SO_4_, 10 mV s^−1^	44
Pinecone	KOH	1 : 1	1515	137	1 M Na_2_SO_4_, 0.1 A g^−1^	45
Elaeocarpus tectorius	H_3_PO_4_	1 : 3.4	858	201	1 M H_2_SO_4_, 1 A g^−1^	29
*Borassus flabellifer* flower	H_3_PO_4_	1 : 4.25	633	238	1 M KOH, 1 A g^−1^	46
Lignocellulose powders	NaH_2_PO_4_ + ZnCl_2_	1 : (0.67 + 1.33)	658	193.6	6 M KOH	47
*Fir sawdust*	H_3_PO_4_	1 : 0.03	1281.6	292 and 169.4 F g^−1^	1 M H_2_SO_4_, 0.1 and 0.5 A g^−1^	This work

Compared with C-*X*, PC-*X* had a higher *C*_g_ capacitance at the corresponding temperature, which indicates that the electrochemical performance of porous carbon is enhanced after phosphorus doping. The doping of the P-functional group can improve the wettability of the electrode and directly generate a pseudocapacitance through a redox reaction. This reaction is presented later.^29,48^
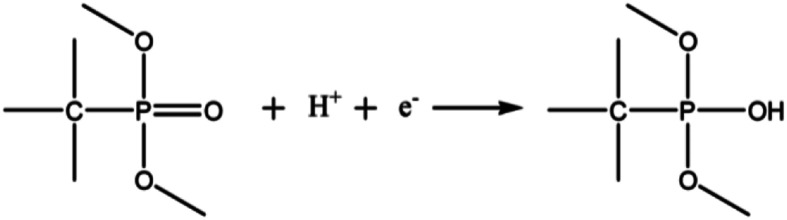


To further understand the capacitive behavior, an electrochemical impedance spectroscopy (EIS) was tested. The Nyquist spectra of C-*X* and PC-*X* are shown in Fig. 7f. As shown in Fig. 7f, the EIS curves of C-*X* and PC-*X* were similar, consisting of a semicircle at high frequencies and a linear component at low frequencies. The line represented Warburg diffusion resistance, which comes from the ion diffusion of the electrolyte. It is generally accepted that the more perpendicular the straight line, the better the capacitive performance of porous carbon.^3^ Thus, at the corresponding temperature, the perpendicularity of PC-*X* is higher than that of C-*X*, which indicates that PC-*X* has better capacitance performance. A semicircle indicates the charge transfer resistance from electrode/electrolyte interface, whereas a smaller circle signifies a faster charge transfer rate.^49^ The presence of small semicircles reveals the pseudocapacitive behavior of porous carbon. Nian *et al.* pointed out that the semicircle of pseudocapacity was due to the interfacial redox reactions of impurities and surface functional groups.^50^ As shown in Fig. 7f, the semicircle of PC-*X* was smaller than that of C-*X*, which further indicates that the capacitance performance of PC-*X* is better. Compared with other electrodes, the vertical line of the PC-900 electrode was the largest, and the semicircle was the smallest, indicating that its diffusion and transfer resistance are lower.

**Fig. 8:**
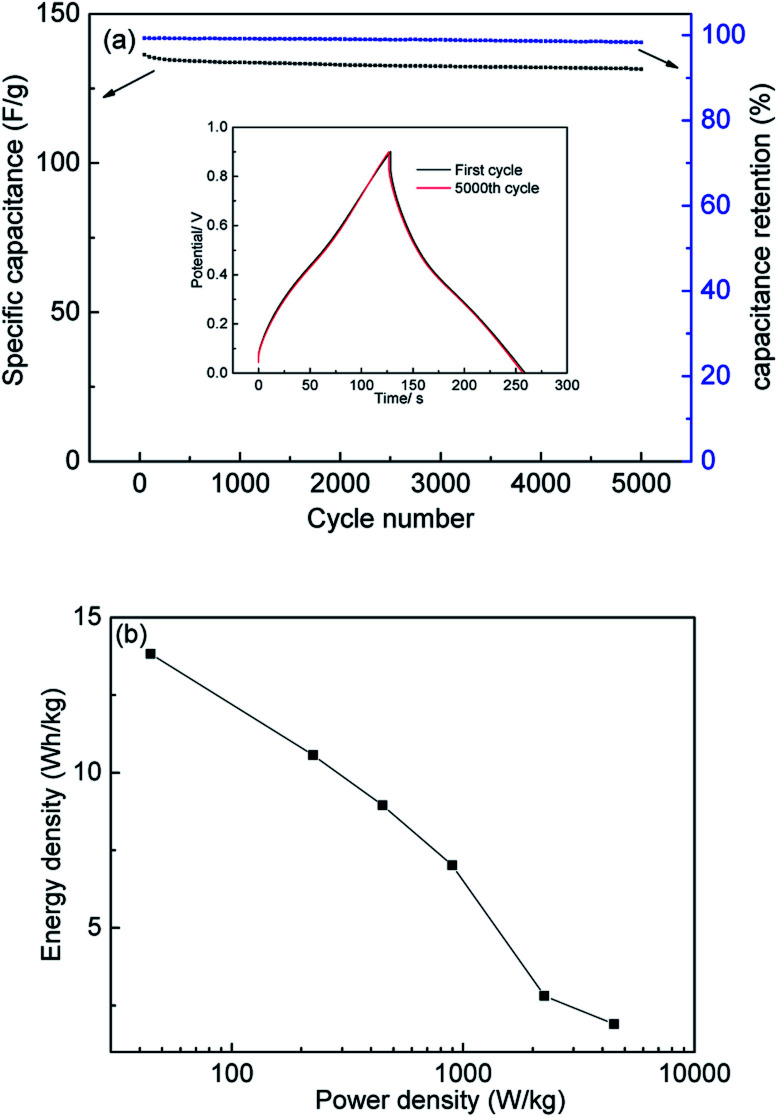
(a) specific capacitances and capacitance retention of PC-900 at 2A g^−1^ (the inset are the first cycle and the 5000^th^ cycle of GCD of PC-900 at 2A g^−1^), (b) Ragone plots of PC-900.

Cycle stability is the most important feature determining whether or not a material can be put to practical use.^51^ The long-term cycle stability of PC-900 was evaluated under the high current density of 2 A g^−1^. After 5000 charge–discharge cycles, it showed excellent cycle stability with a capacitance retention rate of 98.3% (Fig. 8a). The inset of Fig. 8a shows the first and last cycles of the GCD curve, and they still show a symmetrical triangle shape during the cycle test, which indicates excellent electrochemical cycle stability.

Energy density and power density are two practical parameters for evaluating supercapacitors, and they were calculated based on the GCD of a two-electrode system (Fig. S4†). The energy density and power density of PC-900 were calculated and summarized in the Ragone plot (Fig. 8b). As shown in Fig. 8b, the PC-900 based supercapacitor showed a high energy density of 10.6 W h kg^−1^ when the power density was 224.8 W kg^−1^ at the discharging current density of 0.5 A g^−1^. The obtained results show that the potential applications of PC-900-based supercapacitors in energy storage equipment are broad.

## Conclusions

In short, this paper used a small amount of phosphoric acid to impregnate naturally rich biomass sawdust as a carbon precursor and prepare phosphorus-doped porous carbon by a one-step carbonization process. The carbon yield of PC-*X* treated with H_3_PO_4_ was higher than that of untreated C-*X*. In addition, it has been demonstrated that phosphorus having dual properties such as dopant and gas expanding agent plays a vital role in forming uniform micropores and increasing the interlayer distance of carbon, resulting in the formation of excellent EDLC. The obtained carbon PC-900 has a high surface area, a narrow pore size distribution, and phosphorus atoms doped in the carbon material skeleton, which gives it excellent capacitive performance. The specific capacitances of PC-900 reached the maximum values of 292 F g^−1^ and 169.4 F g^−1^ at the current density of 0.1 A g^−1^ and 0.5A g^−1^. Additionally, the porous carbon-based supercapacitor was able to provide a high energy density of 10.6 W h kg^−1^ when the power density was 224.8 W kg^−1^ at a discharging current density of 0.5 A g^−1^, and has an excellent cyclic longevity with 98.3% capacitance retention after 5000 cycles. Therefore, this work provides a simple, low-cost, and environmentally friendly design approach to the preparation of phosphorus-doped porous carbon for energy storage applications.

## Conflicts of interest

There are no conflicts to declare.

## Supplementary Material

RA-010-D0RA02398A-s001
