# Supplementary material for: Preparation of phosphorus-doped porous carbon for high performance supercapacitors by one-step carbonization
Source: RSC Adv. 2020 May 6;10(30):17768–76. doi: 10.1039/d0ra02398a (PMC9053633; doi:10.1039/d0ra02398a)
Supplement: RA-010-D0RA02398A-s001 [file RA-010-D0RA02398A-s001.pdf]

This journal is © The Royal Society of Chemistry 2020

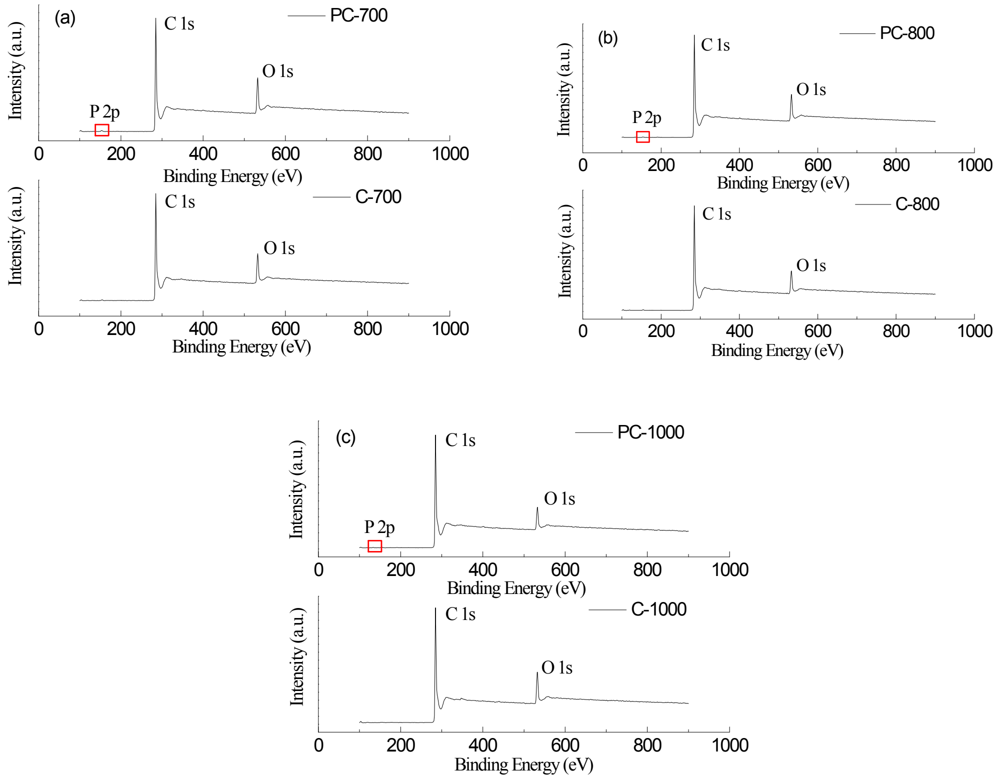

Fig. S1. XPS spectrum of a) C-700 and PC-700, b) C-800 and PC-800, c) C-1000 and PC-1000.

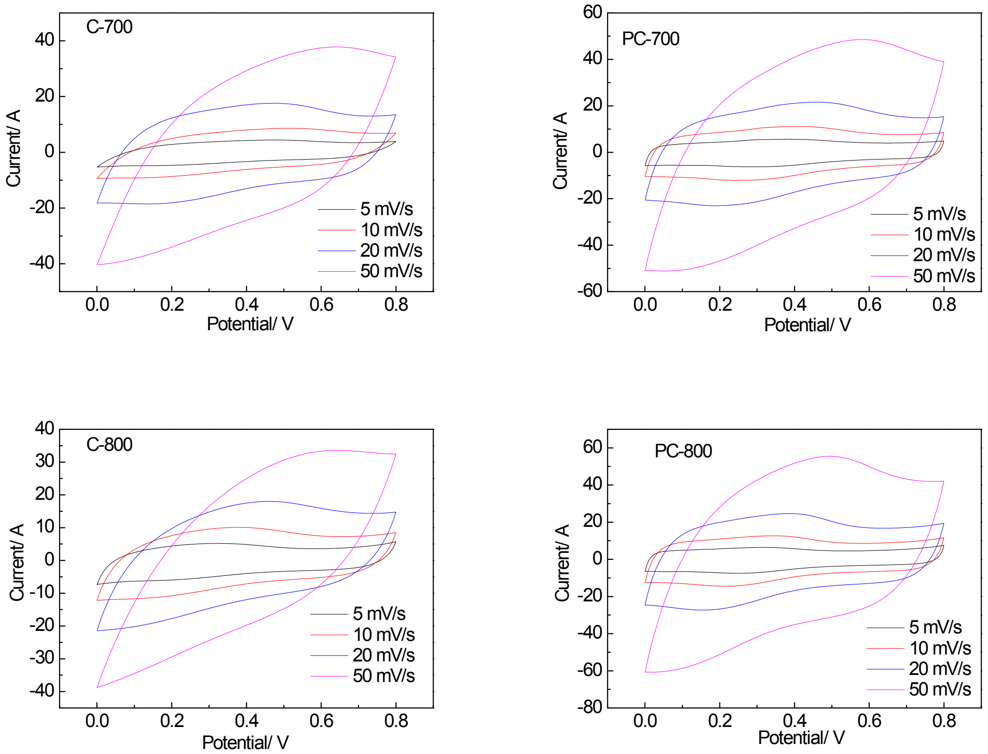

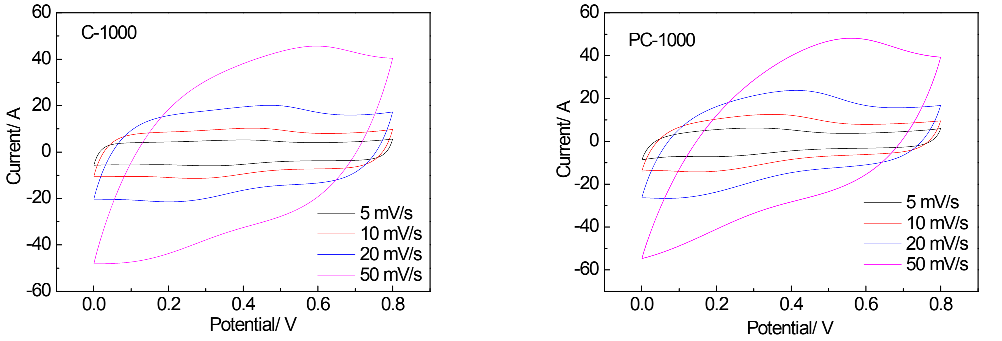

Fig. S2. CV curves of C-700, PC-700, C-800, PC-800, C-1000 and PC-1000.

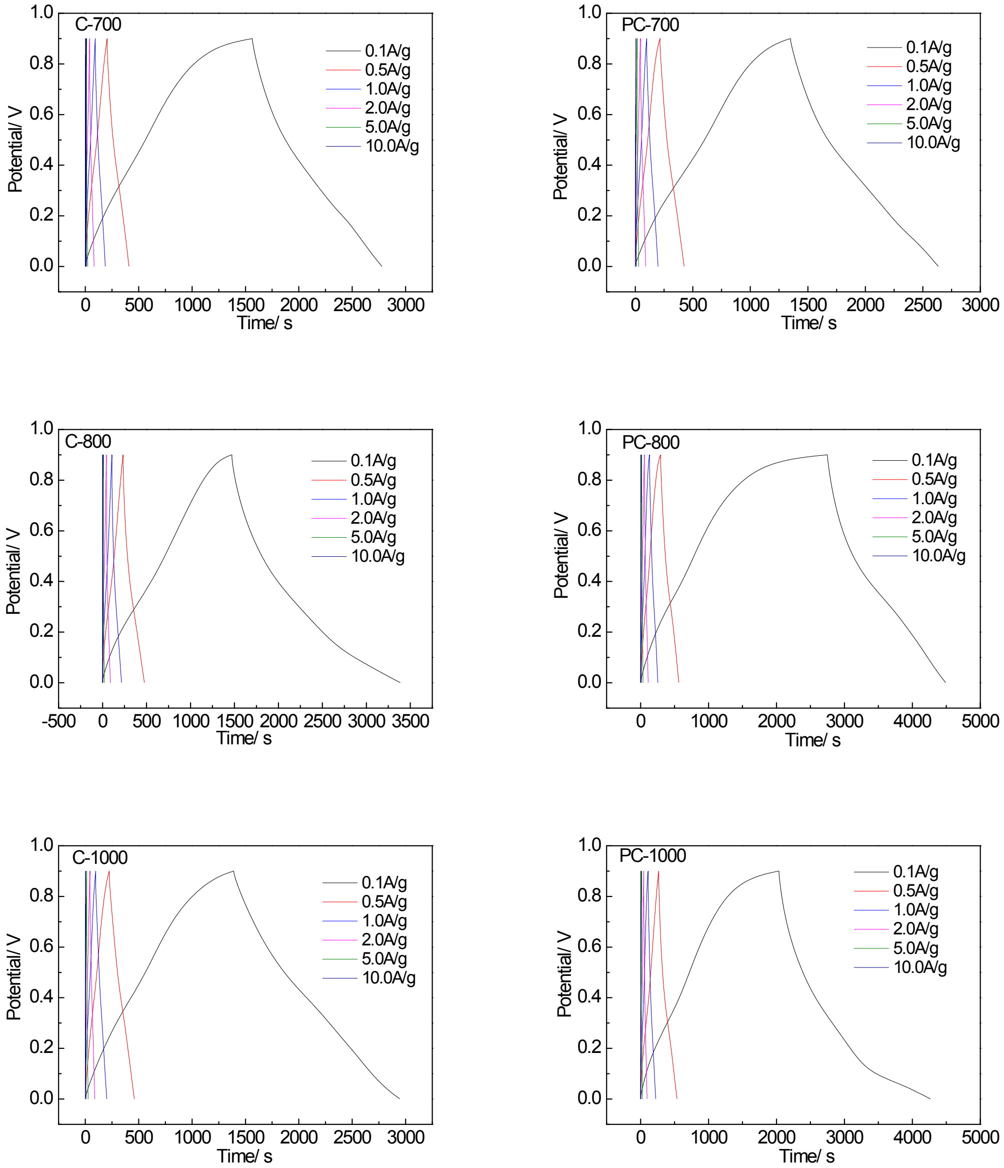

Fig. S3. GCD curves of C-700, PC-700, C-800, PC-800, C-1000 and PC-1000.

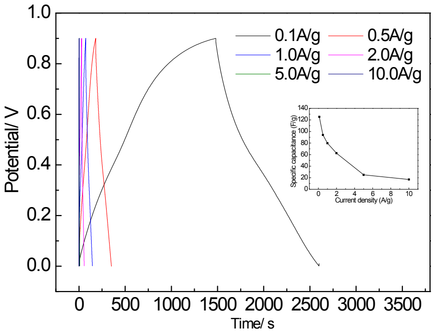

Fig. S4. GCD curves of PC-900 measured on two-electrode system under different current densities. Inset is the specific capacitances of PC-900 at different current densities.
